# The role of preoperative lung rehabilitation training combined with nutritional intervention on surgical tolerance and accelerated recovery indicators in patients with moderate to severe COPD complicated with lung cancer

**DOI:** 10.3389/fsurg.2025.1667085

**Published:** 2025-11-19

**Authors:** Yan Hu, Qin Cheng, Qiucheng Guo

**Affiliations:** Department of Thoracic Surgery, Hubei Cancer Hospital, Tongji Medical College, Huazhong University of Science and Technology, Wuhan, Hubei, China

**Keywords:** nutritional intervention, preoperative lung rehabilitation training, COPD complicated with lung cancer, pulmonary function, surgical tolerance

## Abstract

**Background:**

Lung cancer and chronic obstructive pulmonary disease (COPD) commonly coexist simultaneously and individuals with COPD are at a higher risk of developing lung cancer. Nutritional intervention has a restorative effect on COPD combined with lung cancer, but there is almost no research on lung rehabilitation training, and there are even fewer studies on the combination of the two.

**Objectives:**

Our study aimed to assess the role of preoperative lung rehabilitation training plus nutritional intervention on surgical tolerance and accelerated recovery indicators in patients with moderate to severe COPD complicated with lung cancer.

**Methods:**

A total of 92 patients with COPD complicated by lung cancer who underwent surgery at our hospital between February 2023 and March 2024 were enrolled. Using a block randomization method, patients were divided into two groups: the control group (*n* = 47) receiving only nutritional intervention, and the observation group (*n* = 45) receiving a combination of lung rehabilitation training and nutritional intervention. The following indicators were compared between the two groups: pulmonary function parameters [forced expiratory volume in 1 s (FEV1), FEV1/forced vital capacity (FVC), maximum voluntary minute ventilation percentage (MVV%), and lung carbon monoxide diffusion capacity (DLCO)], Modified Medical Research Council (MMRC) dyspnea scale score, 6 min walking distance (6MWD), blood gas indicators [arterial partial pressure of oxygen (PaO_2_) and arterial partial pressure of carbon dioxide (PaCO_2_)], quality of life, and postoperative complication rate.

**Results:**

After intervention, the observation group showed significantly higher levels of FEV1, FEV1/FVC, MVV%, DLCO, and 6MWD compared with the control group, while the MMRC score was significantly lower (all *p* < 0.05). Regarding blood gas indicators, the observation group had a significantly higher PaO_2_ level and a significantly lower PaCO_2_ level than the control group (*p* < 0.05). Additionally, the quality of life score in the observation group was significantly higher, and the postoperative complication rate was significantly lower than those in the control group (both *p* < 0.05).

**Conclusion:**

Preoperative lung rehabilitation training combined with nutritional intervention can effectively improve pulmonary function and respiratory function in patients with moderate to severe COPD complicated by lung cancer, enhance their surgical tolerance, improve quality of life, and reduce the incidence of postoperative complications.

## Introduction

As the most common malignant tumor, lung cancer continues to increase its incidence rate and mortality, and currently ranks first in cancer deaths (1–3). With the continuous aggravation of air pollution and the induction of risk factors such as long-term smoking, patients with lung cancer combined with COPD are becoming increasingly common in clinical practice, especially those with moderate to severe obstructive ventilation dysfunction, who lose their best surgical opportunities due to poor lung function reserve (4–6). Video assisted thoracoscopy surgery (VATS) has the characteristics of minimal trauma and short postoperative recovery time, making it an ideal surgical method for lung cancer complicated with COPD, which can promote rapid recovery of patients during the perioperative period (7, 8). Nevertheless, surgical treatment for patients can cause metabolic hyperactivity in the body, greatly reducing the synthesis of carbohydrates, fats, and proteins, resulting in poor nutrition and immune regulation disorders in patients (9). Hence, various intervention methods are often used in clinical to improve this situation (10). Nutritional intervention is simple and easy to implement, convenient to operate, but the focus is relatively single, resulting in poor overall efficacy and affecting the treatment process and prognosis (11, 12). Preoperative lung rehabilitation training plays an important role in the prevention of cancer and the treatment of pulmonary disease complications, but there are few reports on the impact on COPD complicated with lung cancer (13). A recent systematic review and meta-analysis of randomized controlled trials confirmed that preoperative pulmonary rehabilitation can significantly improve perioperative outcomes (e.g., pulmonary function, exercise tolerance) in lung cancer patients (14). Additionally, a scoping review on prehabilitation in the lung cancer pathway emphasized that combined interventions (e.g., pulmonary rehabilitation + nutritional support) may yield greater benefits than single strategies, though data on COPD-complicated lung cancer remain limited (15). Hence, this study aimed to discuss the role of preoperative lung rehabilitation training plus nutritional intervention on surgical tolerance and accelerated recovery indicators in patients with moderate to severe COPD complicated with lung cancer.

## Materials and methods

### General data

The clinical data of 92 patients who diagnosed with COPD complicated with lung cancer at our hospital between February 2023 and March 2024 were analyzed, adopting the method of block randomization. All patients were diagnosed with lung cancer through preoperative biopsy, and moderate to severe COPD was defined when the FEV1/FVC < 70%, −2.51 < *Z*-value <−4.00 is moderate, and *Z*-value < −4.10 is severe. there were no statistically significant differences between the two groups in terms of gender, age, body mass index, surgical selection, pathological type, and TNM staging (*p* > 0.05), as shown in Table 1. This study was approved via the Ethics Committee of our hospital.

**Table 1:** General information of the patient.

Characteristics	Observation group (*n* = 45)	Control group (*n* = 47)	*t*/*X*^2^	*p*
Sex				
Male (%)	28 (62.22)	27 (57.45)		
Female (%)	17 (37.78)	20 (42.55)	0.218^b^	0.641
Age (year)	59.53 ± 8.26	59.72 ± 8.13	0.088^a^	0.930
Body mass index (kg/m^2^)	20.34 ± 2.11	20.16 ± 2.37	0.390^a^	0.698
Surgical selection				
Segmentectomy (%)	22 (48.89)	21 (44.68)		
Lobectomy (%)	23 (51.11)	26 (55.32)	0.164^b^	0.686
GOLD				
GOLD1 level	3 (6.67)	2 (4.26)		
GOLD2 level	18 (40.00)	20 (42.55)		
GOLD3 level	22 (48.89)	19 (40.43)		
GOLD4 level	2 (4.44)	6 (12.77)	0.260^b^	0.610
Pathological type				
Squamous cell carcinoma (%)	27 (60.00)	26 (55.32)		
Adenocarcinoma (%)	18 (40.00)	21 (44.68)	0.206^b^	0.650
TNM staging				
I (%)	29 (64.44)	27 (57.45)		
II (%)	16 (35.56)	20 (42.55)	0.473^b^	0.492

a Unpaired Student's *t* test.

b *X*^2^ test.

### Inclusion and exclusion criteria

Inclusion criteria: (1) Diagnosed with COPD complicated with lung cancer through pathological examination; (2) The patient's clinical data is complete; (3) The patient has no mental cognitive impairment; (4) All patients have signed informed consent.

Exclusion criteria: (1) The patient has malignant tumors in other parts of the body; (2) Patient's tumor has distant metastasis; (3) Severe impairment of liver, liver, and kidney function; (4) The patient has a mental illness.

### Treatment method

The control group was treated with nutritional intervention: Dietitian provide nutritional guidance and dietary matching to each patient, and calculate the patient's daily total energy and intake of various nutrients based on the dietary survey results. The patient's daily energy and protein intake should reach 70% of the target value, with a total energy requirement of 25–30 kcal·kg^−1^·d^−1^ and a protein requirement of 1.5–2.0 g·kg^−1^·d^−1^. The weight is calculated based on the ideal weight: ideal weight (kg) = height (cm) −105. If the patient's independent diet is insufficient, oral enteral nutrition supplement (ONS) can be given. Provide ONS nutrition related health education 1d before surgery, and provide personalized guidance according to the patient's chosen surgical sequence. Start taking ONS orally 10 h before surgery, 200–300 ml each time, 2–4 times in total, and stop taking it 2 h before surgery. If the ONS is not well executed and the dietary survey shows that the dietary intake for 7 consecutive days does not reach 60% of the energy intake standard, the clinical physician and nutritionist will discuss the nutritional support plan, and combine the patient's wishes. Decide whether to provide enteral nutrition or parenteral nutrition support through tube feeding. The observation group was treated with lung rehabilitation training + nutritional intervention on the basis of the control group. The specific treatment is as follows: (1) Breathing training: Keep the patient naturally relaxed, take a slow deep breath and hold it for about 5 s. At the end of the deep breath, slowly exhale through the mouth and inwardly contract the abdomen. Train 2–3 times a day for about 15 min each time. (2) Coughing and expectoration training: While taking deep breaths, cross your hands in front of your chest and exhale continuously in large mouthfuls. When the phlegm accumulates in your throat, cough it up vigorously and gently tap the patient's back if necessary to help expel the phlegm. (3) Respiratory gymnastics training: Guide patients to perform appropriate limb training on the basis of deep breathing, including abduction and chest expansion of both arms, lifting during inhalation, etc. At the same time, select aerobic training with appropriate intensity for patients, such as walking, skipping rope, etc., adjust according to the patient's physical condition, about 10 min each time, and train 2–3 times/d. (4) Stair climbing training: Accompanied by medical staff, exercise by pursed lip breathing and exhaling with force. Depending on the individual's condition, if there is slight wheezing, continue. If there is obvious difficulty breathing, take a short break before continuing, 15–30 min/time, twice a day. (5) Weightlifting training: Patients lift objects weighing 0.5–3 kg above their head and shoulders for 10–15 min each time, twice a day.

### Observation indicators

The observation indicators were as follows: (1) Pulmonary function indicators: Before and after 4 weeks of intervention, compare the lung function indicators of the two groups of patients, including forced expiratory volume in one second (FEV1), forced vital capacity (FVC), MVV (Maximum autonomous minute ventilation volume), DLCO (diffusion capacity of the lung for carbon monoxide). (2) 6 min walking distance (6MWD): The six minute walking test was used to evaluate the exercise tolerance of two groups of patients. The test was conducted in a quiet, safe, and well ventilated corridor of 30 m. Before the test, the testing method was demonstrated to the patients, and records were kept. If breathing difficulties, chest pain, or other conditions occurred during the process, the test should be terminated and the cause identified, and symptomatic treatment should be given. (3) The modified dyspnea scale(mMRC): The mMRC scale was used to evaluate respiratory distress in both groups. It classifies respiratory distress into 5 levels (0–4), with higher scores indicating more severe dyspnea. (4) Blood gas indicators: Before and after 4 weeks of intervention, compare the blood gas indicators of two groups of patients, including arterial partial pressure of oxygen (PaO_2_) and arterial partial pressure of carbon dioxide (PaCO_2_), the above indicators were detected using ABL800FLEX blood gas analyzer. (5) Quality of life: The European Organization for the Treatment of Cancer Quality of Life Scale (EORTC QLQ-C30) was used to evaluate the quality of life of patients, including functional areas, general health, and symptom areas. The higher the score, the better the quality of life. (6) Incidence of complications: Compared the incidence of postoperative complications between two groups of patients, including pulmonary infection, dyspnea, atelectasis, and pulmonary leakage.

### Statistical processing

Data were analyzed using SPSS 25.0 software. Measurement data were expressed as mean ± standard deviation (*x* ± *s*), and inter-group comparisons were conducted using the *t*-test. Counting data were presented as rate, and inter-group comparisons were performed using the *χ*^2^ test. *P* < 0.05 was considered statistically significant.

## Results

### Multidimensional outcome indicators of the comprehensive efficacy of intervention measures

Prior to intervention, there were no significant differences between the two groups in 6 min walk distance (6MWD), mMRC dyspnea scores, blood gas parameters (PaO_2_, PaCO_2_), or quality of life scores (all *p* > 0.05), indicating comparable baseline characteristics. Post-intervention, both groups showed significant improvements in the aforementioned indicators compared to pre-intervention (all *p* < 0.01). However, intergroup comparisons revealed that the observation group demonstrated significantly greater improvement than the control group across all measures. Specifically: the observation group demonstrated significantly longer 6MWD, significantly lower mMRC scores, higher PaO_2_ levels, lower PaCO_2_ levels, and significantly higher scores across all four quality-of-life dimensions (physical, emotional, social, and cognitive) compared to the control group (all *p* < 0.01). Detailed data are presented in Table 2.

**Table 2:** Comparison of preoperative and postoperative pulmonary function, exercise capacity, blood gas indicators and quality of life between the two groups of patients $\left(\bar{x}\pm s\right)$.

Indicator	Group	Preoperative	Postoperative	*p* (Within group)	*p* (Between groups, preop	*p* (Between groups, postop)
Pulmonary Function						
FEV1 (%)	Observation *n* = 45	64.29 ± 10.25	78.34 ± 9.26^*#^		0.940	<0.0001
	Control *n* = 47	64.13 ± 10.36	71.29 ± 9.48*			
FEV1/FVC	Observation *n* = 45	63.15 ± 9.46	77.29 ± 9.31^*#^		0.910	<0.0001
	Control *n* = 47	63.37 ± 9.25	70.25 ± 9.16*			
MVV%	Observation *n* = 45	47.25 ± 5.13	70.34 ± 5.64^*#^		0.918	<0.0001
	Control *n* = 47	47.36 ± 5.29	64.35 ± 5.72*			
DLCO	Observation *n* = 45	2.13 ± 0.34	2.56 ± 0.27^*#^		0.544	0.002
	Control *n* = 47	2.17 ± 0.31	2.38 ± 0.29*			
Exercise Capacity						
6MWD (m)	Observation *n* = 45	318.46 ± 62.57	664.21 ± 63.18^*#^		0.935	<0.0001
	Control *n* = 47	319.52 ± 62.31	532.47 ± 62.96*			
MMRC scores	Observation *n* = 45	2.25 ± 0.43	1.52 ± 0.28^*#^		0.810	<0.0001
	Control *n* = 47	2.27 ± 0.38	2.03 ± 0.26*			
Blood Gas Analysis						
PaO_2_ (mmHg)	Observation *n* = 45	66.47 ± 10.25	85.26 ± 11.34^*#^		0.563	<0.0001
	Control *n* = 47	65.23 ± 10.48	75.32 ± 10.69*			
PaCO_2_ (mmHg)	Observation *n* = 45	43.15 ± 4.58	31.62 ± 4.53^*#^		0.859	<0.0001
	Control *n* = 47	42.98 ± 4.63	38.36 ± 4.67*			
Quality of Life						
Physical function	Observation *n* = 45	60.32 ± 6.15	78.45 ± 6.21^*#^	<0.01	0.864	<0.0001
	Control *n* = 47	60.54 ± 6.27	69.26 ± 6.53*	<0.01		
Emotional function	Observation *n* = 45	58.42 ± 4.63	76.21 ± 4.12^*#^	<0.01	0.958	<0.0001
	Control *n* = 47	58.37 ± 4.55	64.26 ± 4.29*	<0.01		
Social function	Observation *n* = 45	60.13 ± 4.28	77.43 ± 5.13^*#^	<0.01	0.876	<0.0001
	Control *n* = 47	60.27 ± 4.35	68.36 ± 5.24*	<0.01		
Cognitive function	Observation *n* = 45	61.32 ± 5.48	81.25 ± 5.32^*#^	<0.01	0.978	<0.0001
	Control *n* = 47	61.29 ± 5.37	69.43 ± 5.16*	<0.01		

* *p* < 0.01 compared to preoperative in the same group.

# *p* < 0.01 compared to the control group at the same time point (postoperative).

6MWD, 6-minute walking distance; MMRC, the modified medical research council dyspnea scale; PaO_2_, arterial partial pressure of oxygen; PaCO_2_, arterial partial pressure of carbon dioxide.

### Incidence of complications

After intervention, The incidence of complications in the observation group (13.33%) was lower than that in the control group (31.91%, *p* < 0.05), as shown in Table 3.

**Table 3:** Comparison of postoperative complications between two groups (*n*, %).

Group	*n*	Number of pneumonia (%)	Number of atelectasis (%)	Number of pneumothorax (%)	Number of pulmonary abscess (%)	Overall incidence rate (%)
Observation group	45	1	2	2	1	6 (13.33)
Control group	47	2	3	7	3	15 (31.91)
*X* ^2^						4.506
*p*						0.034

## Discussion

Lung cancer is one of the cancers with a high mortality rate worldwide, and its causes are mainly related to smoking, occupational exposure, environmental pollution, genetics, and other factors (16, 17). Research has shown that the incidence of lung cancer complicated with COPD is 10.8%, and lung cancer is the main cause of death for COPD patients, with approximately 4%–33% of COPD patients dying from lung cancer (18, 19). Thus, with the trend of population aging and the impact of environmental pollution, the number of lung cancer patients with COPD is increasing year by year (20). Video assisted thoracoscopy (VATS) is a minimally invasive surgery that can effectively remove tumor lesions (21). Compared with traditional surgery, it has the characteristics of less trauma, faster postoperative recovery, and lower complications (22). It is an ideal surgical method for COPD combined with lung cancer (23). However, due to the decrease in lung capacity and ventilation, the oxygen utilization rate decreases, and the ability of the lungs to clear secretions decreases, resulting in an increase in the viscosity of respiratory secretions, which seriously affects the patient's recovery and reduces their quality of life (24, 25). Therefore, it is particularly important to take effective intervention measures for COPD combined with lung cancer patients to improve their lung function and enhance their quality of life.

Early nutritional intervention can provide patients with the necessary nutrients for survival, maintain a state of metabolic balance, and improve their nutritional indicators (26). However, nutritional interventions lack personalization and specificity, making it difficult to achieve ideal improvement effects on patients (27). Preoperative pulmonary rehabilitation training is an intervention measure aimed at patients with respiratory system diseases (28). Through training of limb skeletal muscles, lung capacity, and respiratory muscles, it alleviates patients' respiratory distress symptoms and improves the recovery of limb function (29). It is a comprehensive and scientific non pharmacological intervention method (30). A study has found that preoperative lung rehabilitation training could improve lung function and prolong survival time in lung cancer patients (31). Howbeit, there is limited research on the effects of preoperative lung rehabilitation training combined with nutritional intervention on COPD complicated with lung cancer.

Our study discovered that after 4 weeks of intervention, the FEV1/FVC, MVV%, and DLCO in observation group were significantly higher than those in the control group. Additionally, the level of PaO_2_ in observation group was higher than control group, while the level of PaCO_2_ in observation group was lower than control group, which indicated that preoperative lung rehabilitation training combined with nutritional intervention could help improve active respiratory function after COPD combined with lung cancer surgery. By increasing respiratory muscle exercise, alveolar ventilation function could be improved, thereby increasing lung capacity and improving lung function, which was consistent with the results of Lai et al. (32).

6MWD and MMRC as important indicators for lung cancer rehabilitation assessment (33). The longer the 6MWD, the higher the degree of lung rehabilitation. The higher the MMRC score, the more severe the breathing difficulties (34). Divisi et al. (35) found preoperative respiratory training could improve 6MWD in lung cancer patients and reduce postoperative complications, which was consistent with our results. After 4 weeks of intervention, the 6MWD was higher in observation group than in control group, while the MMRC scores in observation group was lower than control group. There were two main reasons. Firstly, Preoperative pulmonary rehabilitation training combined with nutritional intervention could effectively improve ventilation volume, increase patient surgical tolerance, eliminate residual gas, and reduce dead space ventilation (36). Secondly, this intervention method was able to improve respiratory muscle strength and exercise tolerance, thereby reducing postoperative respiratory distress and alleviating (37). What's more, the EORTC QLQ-C30 scores in all dimensions of the observation group were higher than control group, while the incidence of adverse events was lower than control group, which implied that preoperative lung rehabilitation training plus nutritional intervention could promote sufficient lung expansion, further clear lung cancer airway secretions and sputum, accelerate patient recovery, and lessen the occurrence of complications. Laurent et al. (38) also discovered the same result.

Yet, this study has several limitations that need to be acknowledged. First, as a single-center study, the sample may lack representativeness, and results may not be generalized to other populations. Second, the follow-up duration was short (4 weeks post-intervention), and we did not assess long-term outcomes such as progression-free survival (PFS) and overall survival (OS)—key indicators for evaluating the intervention's impact on patient prognosis. Third, although block randomization balanced baseline characteristics (e.g., gender, age, BMI), potential confounding factors were not fully adjusted for: (1) Baseline exercise tolerance (a key predictor of pulmonary rehabilitation response) was not measured; (2) Tumor staging was simplified to I/II (no sub-staging, e.g., T1a/T1b), which may correlate with surgical difficulty; (3) Surgical details (e.g., extent of resection) were not recorded. Fourth, the small sample size (92 patients) reduces statistical power to detect subtle between-group differences. In the future, multi-center studies with larger samples, extended follow-up (to assess PFS/OS), and adjustment for confounding factors (e.g., baseline exercise tolerance) are needed to validate our findings.

All in all, preoperative pulmonary rehabilitation training plus nutritional intervention was able to ameliorate lung function, enhance surgical tolerance, improve patients' quality of life, and reduce the occurrence of complications in COPD combined with lung cancer.

## Data Availability

The raw data supporting the conclusions of this article will be made available by the authors, without undue reservation.
